# Short-term moderate caloric restriction in the rhesus macaque attenuates markers of ovarian aging in select populations

**DOI:** 10.18632/aging.206253

**Published:** 2025-05-20

**Authors:** Emma S. Gargus, Rhea Sharma, Rebecca Gu, Camille Mulcahy, Brian W. Johnson, Jing Song, Jungwha Lee, Mary Zelinski, Francesca E. Duncan

**Affiliations:** 1 Department of Obstetrics and Gynecology, Feinberg School of Medicine, Northwestern University, Chicago, IL 60611, USA; 2 Department of Comparative Medicine, Histology and Imaging Core, University of Washington, Seattle, WA 98195, USA; 3 Department of Preventive Medicine, Feinberg School of Medicine, Northwestern University, Chicago, IL 60611, USA; 4 Division of Reproductive and Developmental Sciences, Oregon National Primate Research Center, Beaverton, OR 97006, USA; 5 Department of Obstetrics and Gynecology, Oregon Health and Science University, Portland, OR 97239, USA

**Keywords:** ovarian reserve, fibrosis, aging, nonhuman primate, caloric restriction

## Abstract

Ovarian aging results in decreased fertility and endocrine function. In mice, caloric restriction (CR) maintains ovarian function. In this study, we determined whether CR also has a beneficial effect on reproductive longevity in the nonhuman primate (NHP). Ovaries were collected from young (10–13 years) and old (19–26 years) rhesus macaques who were either on a diet of moderate caloric restriction or a control diet for three years. To test the effect of CR on follicle number, follicles were analyzed in histological sections from animals across experimental cohorts: Young Control, Young CR, Old Control, Old CR (*n* = 4–8/group). In control animals, there was an age-dependent decrease in follicle numbers across all follicle stages (*P* < 0.05). Although there was no effect of diet on total follicle number, the follicle distribution in the Old CR cohort more closely resembled that of young animals. The subset of Old CR animals that were still cycling, albeit irregularly, had more primordial follicles than controls (*P* < 0.05). Assessment of collagen and hyaluronic acid matrices revealed that CR attenuated age-related changes to the ovarian microenvironment. Overall, CR may improve aspects of reproductive longevity in the NHP, but the timing of when it occurs during the reproductive lifespan is likely critical.

## INTRODUCTION

The ovary is one of the first organs to age in humans, with a significant decrease in fertility occurring when women reach their mid-30s due to decreased egg quality and continual follicle loss [1]. The age of onset of menopause is determined by the depletion of the ovarian follicle reserve [2], and this has important implications for overall health since follicles produce estrogen which regulates numerous downstream organ systems [3]. In addition to loss of ovarian follicles, ovarian aging is also characterized by fibrosis of the ovarian stroma. Age-related fibrosis, or the thickening and stiffening of the extracellular matrix due to increased collagen and/or decreased hyaluronic acid, has been demonstrated in murine and human ovaries [4, 5]. The biological sequelae of ovarian aging have significant societal and clinical ramifications as increasingly more women are postponing childbearing to older ages, [6] and more women are living longer post-menopause due to health and medical interventions [7]. Beyond physiologic aging, women with a prior history of medical conditions (e.g., prior ovarian surgery, chemotherapy, radiation therapy, severe endometriosis) or lifestyle factors associated with decreased ovarian function (e.g., smoking) are particularly at risk of a premature decline in their ovarian reserve and accelerated reproductive aging [1].

Given the tangible effects of ovarian aging, there is a need to establish interventions that improve reproductive longevity. Caloric restriction (CR) is a paradigm of extending longevity that was first described in the 1930s [8]. CR slows physiologic aging in many species, including fruit flies, fish, rodents, and nonhuman primates [9–15]. CR in mice is associated with other beneficial effects, including decreases in body weight, abdominal visceral fat, and insulin resistance [16]. Importantly, CR may also be a viable strategy to mitigate ovarian aging. The decreased metabolic rate and body weight that occur following CR have been shown to delay reproductive aging [17, 18]. In mice, CR maintains ovarian function into advanced ages, with higher numbers of ovarian follicles, decreased egg aneuploidy, and increased fecundity relative to controls [19]. Levels of 20% and 40% CR in female mice and rats, respectively, preserved the ovarian reserve [16].

Although the findings regarding CR in rodents are promising, they must be validated in a more relevant model prior to clinical translation. The female rhesus macaque (Macaca mulatta) is an ideal model for studying ovarian aging in primates. Rhesus macaques share ~93% of their genomic sequence with humans [20], and the reproductive anatomy and menstrual cycles of female macaques are similar to those of human females [21]. Rhesus monkeys exhibit menstrual cycles comparable to women, both hormonally and with regard to the endometrial sloughing of the uterine lining [22]. Importantly, rhesus macaques experience similar age-related ovarian changes as observed in women and female chimpanzees [23, 24]. Limited data have demonstrated that rhesus monkeys undergo menopause similar to women at approximately 25 years [25]. Unlike other laboratory animal models such as the rat and mouse, rhesus monkeys may be monitored for cessation of menstruation as is practiced clinically with human patients. Rhesus macaques have many similarities to humans, but the ability to define all environmental variables, including diet, is unique to NHP. Nonetheless, there are some differences between primates and humans that may limit translatability. Macaques exhibit seasonal breeding patterns, in which reproductive hormonal levels and gonadal activity are synchronized with environmental cues, such as a photoperiod. The photoperiod or seasonal pattern can also lead to periodic fluctuations in the neuroendocrine system (hypothalamic-pituitary axis), confounding the observed age-related effect on reproductive or endocrine physiology [26–28]. Despite these considerations, NHP remain a valid and relevant model for humans.

CR intervention at a moderate level in rhesus monkeys has resulted in improvement in overall health and survival [20], including decreased fat, improved metabolism, and decreased risk for cardiovascular disease and diabetes [29]. The effect of CR on parameters of ovarian aging in primates, however, has not been systematically investigated. Thus, the goal of this study was to determine whether short-term (3 year) moderate CR administered in young and old rhesus macaques confers a beneficial effect on follicle dynamics and the extracellular matrix composition of the ovarian microenvironment.

## RESULTS

### A nonhuman primate model of short-term moderate CR

**Figure 1:**
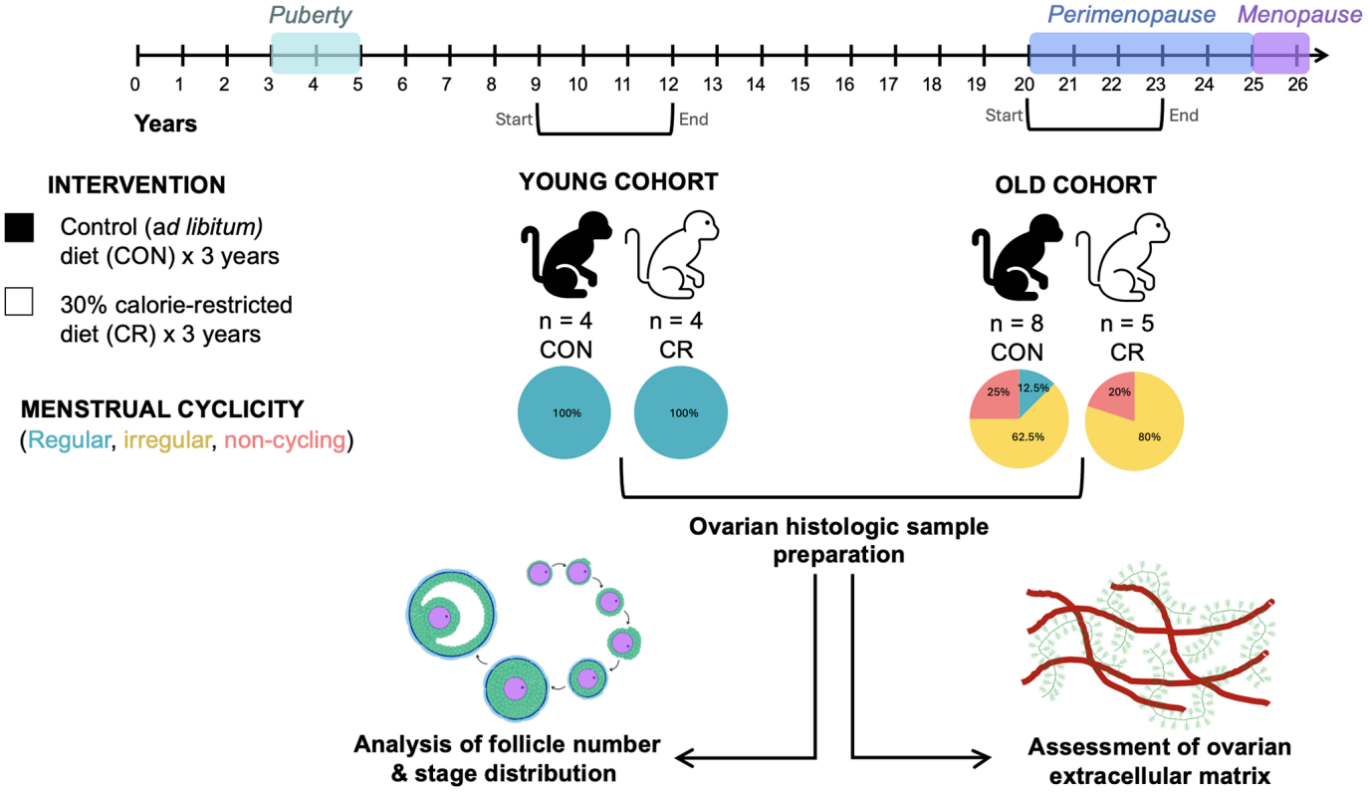
**Schematic of caloric restriction paradigm.** Animals were exposed to a moderate caloric restriction for up to 3 years. Animals in the young cohort were aged 7–10 years at the start of the study and age 10–13 years at necropsy. Animals in the old cohort were aged 16–23 years at the start of the study and age 19–26 years at necropsy. Animals’ menstrual cyclicity was assessed using hormonal assays and observation of menses. Animals were classified as having regular cycles, irregular cycles, or non-cycling. All animals in the young cohort had regular cycles, while animals in the old cohort had regular cycles, irregular cycles, or were non-cycling. Following necropsy, ovarian tissue was harvested and prepared for downstream analyses of ovarian follicle number, follicular dynamics, and extracellular matrix composition. The timeline at the top of the image describes reproductive milestones in the rhesus macaque life cycle. Brackets beneath the timeline indicate the start and end of the study period for the two age cohorts.

To investigate the impact of CR and age on ovarian aging parameters, we acquired a subset of archived ovarian tissue samples from a prior study involving short-term moderate CR in the rhesus macaque in two age cohorts (Figure 1). In the “young” cohort, animals ranged in age from 7–10 years of age at the start of the study, and in the “old cohort” they ranged from 16–23 years of age at the start of the study (Figure 1). The animals were either maintained on a control diet (CON) or one of 30% CR (CR) for a period of three years after which necropsy was performed to harvest organs, including ovaries, for downstream analyses. At the time of necropsy, young animals were 12.3 ± 1.5 (control diet, *N* = 4) and 13.0 ± 0 years of age (CR diet, *N* = 4), and the old animals were 24 ± 2.5 (control diet, *N* = 8) and 23.7 ± 2.6 years of age (CR diet, *N* = 5) (Table 1). There was no significant difference in age at the time of necropsy based on diet group. The period of the study spanned adolescence and reproductive adulthood in the young cohort and reproductive adulthood, perimenopause, and menopause in the old cohort (Figure 1). All young animals in both the CON and CR groups had regular cycles. Old animals were classified as either regular, irregular, or not cycling based on their hormone profile (Figure 1 and Table 1). There were no regularly cycling animals in the old CR group (Figure 1 and Table 1). No differences in estradiol (E2), progesterone (P4), follicle-stimulating hormone (FSH) or luteinizing hormone (LH; relative to the LH peak) were noted among animals with regular cycles between young and old cohorts (data not shown). Thus, it appears that the CR diet permits normal functioning of the reproductive axis in young regular cyclers. By year 3 of the diet, the relative numbers of irregularly cycling and non-cycling animals increased in both the old CON and old CR groups due the animals entering perimenopause (Figure 1 and Table 1). Initiation of CR later in life did not appear to be beneficial to ovarian cyclicity as similar impairments occurred with age in both the old CON and old CR groups (Figure 1 and Table 1), however the small sample limits our ability to draw definitive conclusions.

**Table 1:** Animal and ovarian tissue characteristics.

***n***	**Young CON**	**Young CR**		**Old CON**	**Old CR**	
	4	4		8	5	
**Age at necropsy, years**						
Mean ± S.D.	12.3 ± 1.5	13.0 ± 0.0	*ns*	24.0 ± 2.5	23.7 ± 2.6	*ns*
Range	(10–13)	(13–13)	*p* = 0.36	(19–26)	(20–26)	*p* = 0.86
Menstrual cyclicity	4/4 Regular	4/4 Regular	*ns*	1/8 Regular		*ns*
			*p* > 0.99	5/8 Irregular	4/5 Irregular	*p* = 0.67
				2/8 Non-cycling	1/5 Non-cycling	
**Ovary weight, mg**						
Mean ± S.D.	383.0 ± 130.1	379.8 ± 143.4	*ns*	243.6 ± 94.2	159.6 ± 62.9	*ns*
Range	(314–578)	(205–554)	*p* = 0.97	(145 - 377)	(72–226)	*p* = 0.11
**Histologic slides generated, number**						
Mean ± S.D.	442.5 ± 115.6	347.5 ± 88.5	*ns*	270.0 ± 77.1	278.0 ± 70.9	*ns*
Range	(320–560)	(220–420)	*p* = 0.24	(190–430)	(160–340)	*p* = 0.85

### The age-associated decrease in follicle number is not mitigated by CR, but CR exhibits a more youthful follicle class distribution in aged animals

**Figure 2:**
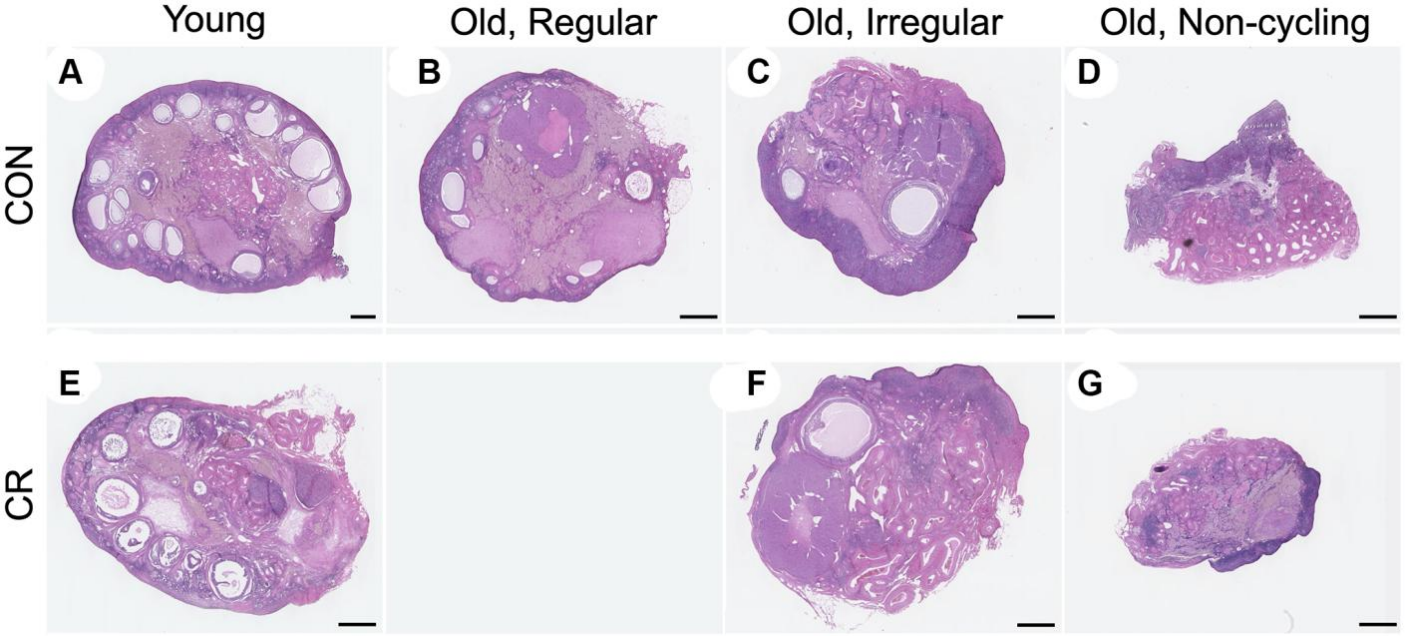
**Representative histologic sections from young, old with regular cycles, old with irregular cycles, and old non-cycling animals exposed to control or calorie-restricted (CR) diet.** Representative midsections from the following cohorts are shown: (**A**) young control, (**B**) old, regular cycling control, (**C**) old, irregular cycling control, (**D**) old non-cycling control, (**E**) young CR, (**F**) old, irregular cycling CR, (**G**) and old non-cycling CR. There were no old, regularly cycling CR animals. The scale bar is 1 mm.

To examine the effect of age and CR on ovarian aging parameters in the nonhuman primate, ovaries were harvested at the time of necropsy. The ovaries from young animals weighed more than those from old animals likely due to the greater number of follicles, but there was no difference within age cohort based on diet (Table 1). Consistent with the age-dependent difference in ovarian weight, we were able to obtain more histologic tissue sections (a surrogate marker for ovarian volume) following serial sectioning of young ovaries relative to old (Table 1). In mammals, the widely held dogma is that females are born with a finite and nonrenewable pool of primordial follicles which comprise the ovarian reserve and dictate reproductive lifespan [30]. These follicles are gradually depleted as females age, and at the time of menopause (cessation of ovarian cycling), very few follicles remain. When examining representative histological sections of the ovaries across experimental cohorts at a gross level, there was an obvious age-dependent decline in the number of visible follicles as expected (Figure 2).

**Figure 3:**
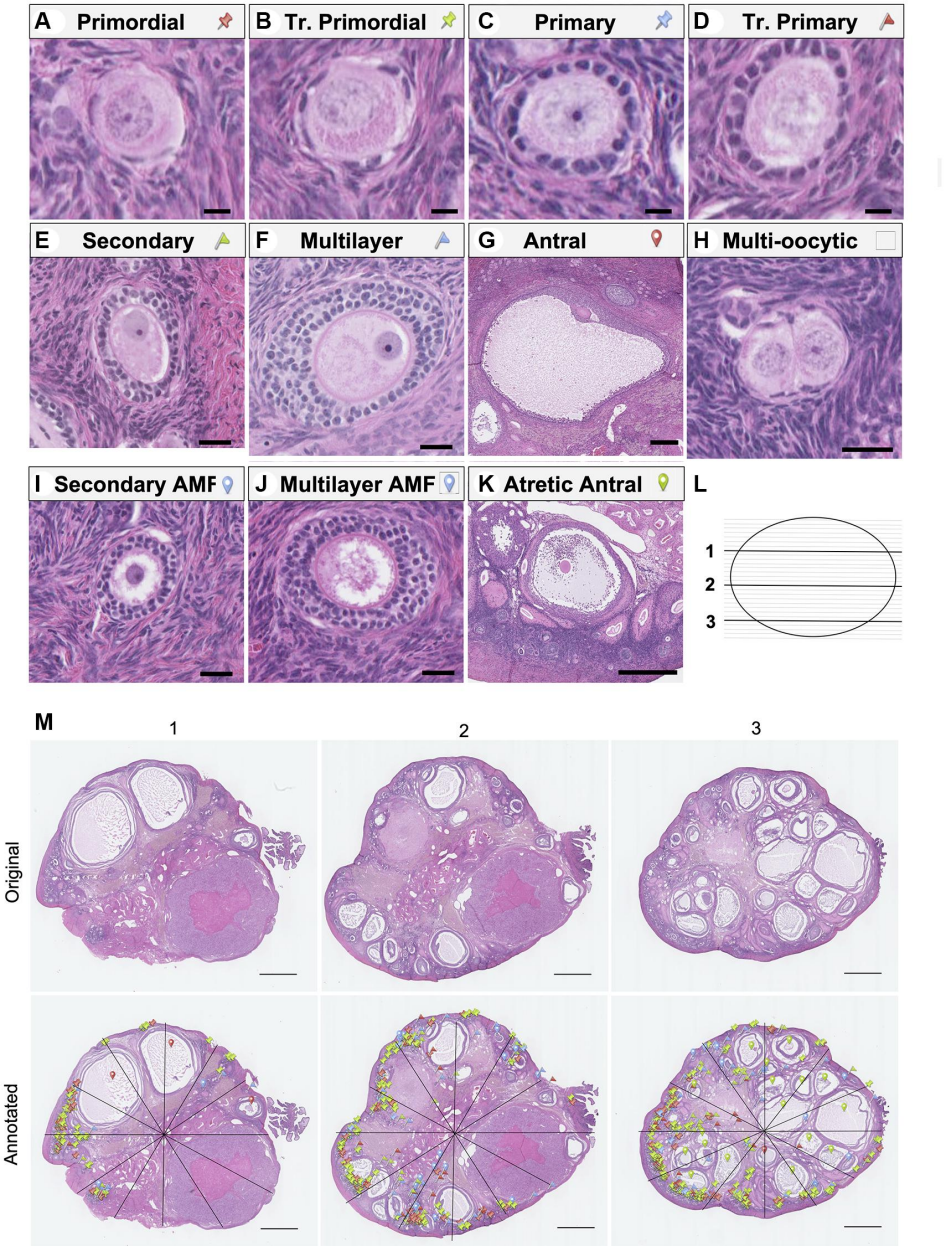
**Representative images of follicle classes and follicle counting method schematic.** Representative hematoxylin-and-eosin-stained images illustrating the following follicle classes are shown: (**A**) primordial, (**B**) transitional primordial), (**C**) primary, (**D**) transitional primary, (**E**) secondary, (**F**) multilayer, (**G**) antral, (**H**) multi-oocytic, (**I**) secondary with abnormal follicle morphology (AMF), (**J**) multilayer with AMF, (**K**) and atretic antral follicles. The follicle counting workflow depicting (**L**) the selection of representative ovarian sections from the top (1), middle (2), and bottom (3) quartiles of the ovary, and (**M**) schematic of follicle counting workflow, including dividing each section into twelve radial segments and annotation with colored markers as defined in A-K to mark and classify each follicle class. The scale bars are 10 μm (**A**–**D**), 25 μm (**E**, **F**, **H**, **I** and **J**), 250 μm (**G**), 500 μm (**K**), and 1 mm (**N**).

**Figure 4:**
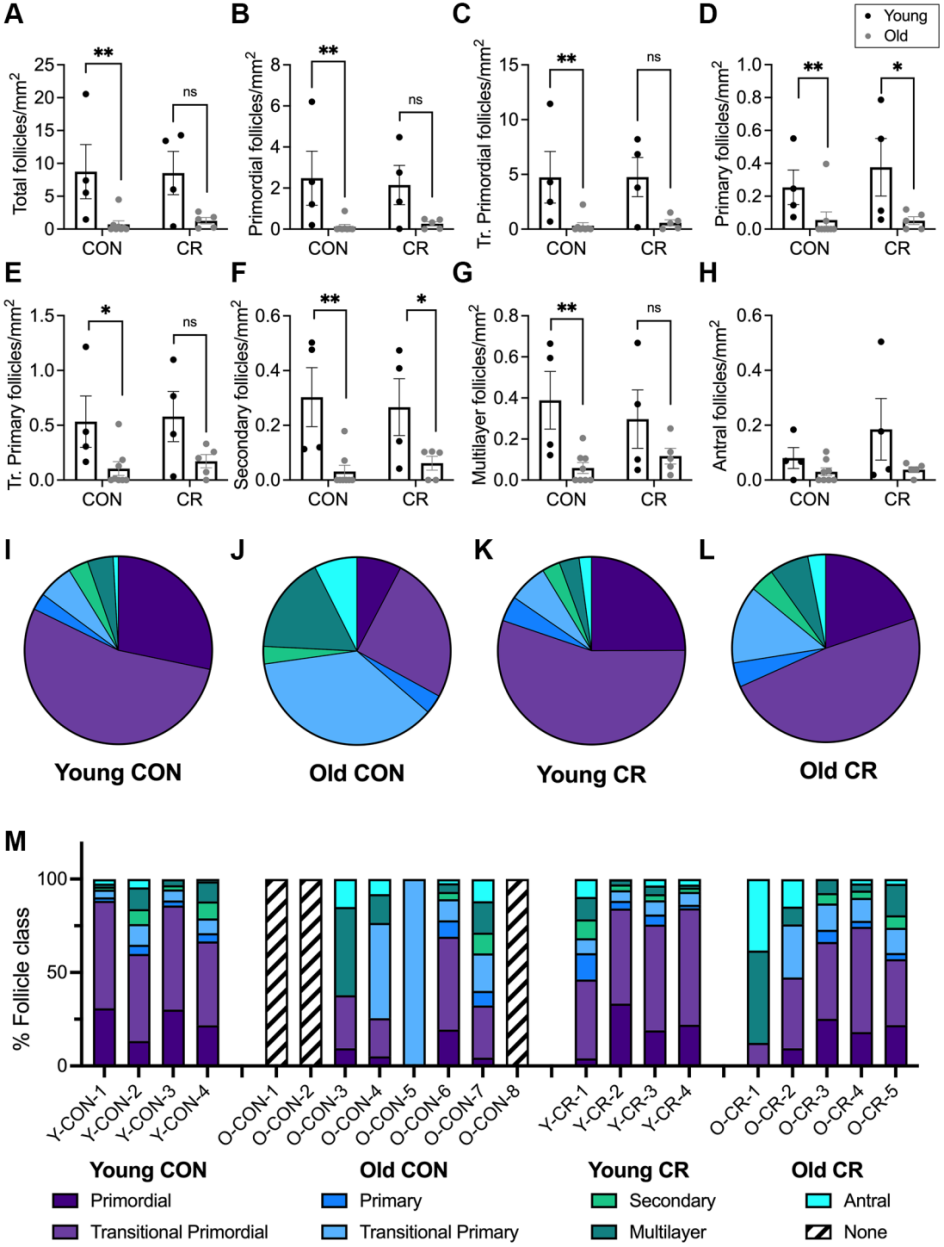
**Follicle number per area differ between young and old animals and follicle class distribution and is affected by age and diet.** Bar graphs of follicle number per ovarian section area for each normal follicle morphology class are shown for: (**A**) total number of follicles, (**B**) primordial, (**C**) transitional primordial, (**D**) primary, (**E**) transitional primary, (**F**) secondary, (**G**) multilayer, and (**H**) antral follicles. Pie charts show follicle class distribution for (**I**) young control, (**J**) old control, (**K**) young calorie-restricted, and (**L**) old calorie-restricted animals. Pie charts were generated by summing raw follicle number from each follicle class for all animals in an experimental group ((**I**) *N* = 4 animals, *n* = 3315 follicles, (**J**) *N* = 5 animals, *n* = 467 follicles, (**K**) *N* = 4 animals, *n* = 3448 follicles, and (**L**) *N* = 5 animals, *n* = 336 follicles) and plotted as pie charts to show follicle distribution across developmental classes. Data are shown as follicle number from each class summed for all animals in the given group (young control, young calorie-restricted, old control, old calorie-restricted). (**M**) follicle class distribution (shown as a percent) for individual animals. Animal identification codes are listed below the X-axis in panel M and can be cross-referenced to additional animal characteristics in Supplementary Table 2. Follicle number per area is plotted as untransformed data. Data are presented as mean ± SEM. Black circles indicate young animals, grey circles indicate old animals. Statistics were calculated on log-transformed data with a two-way ANOVA. Tukey’s post hoc test was applied when one or both of the main effects were statistically significant. ^*^p ≤ 0.05, ^**^p ≤ 0.01. *P*-values for sources of variation of two-way ANOVA can be found in Supplementary Table 1.

To gain further insight into how follicle dynamics in the rhesus macaque are impacted by age and diet, we classified and counted all the major follicle stages with normal morphology in the ovary (Figure 3). These included the quiescent pool of primordial follicles (Figure 3A) as well as those that had activated and were growing, including transitional primordial, primary, transitional primary, secondary, multilayer, and antral follicles (Figure 3B–3G). In addition to healthy follicles, we also classified and counted multi-oocytic follicles, secondary and multilayer follicles with abnormal morphological features, and atretic antral follicles (Figure 3H–3K). For each animal across experimental cohorts, we performed follicle counts in three histologic sections that spanned the ovary (Figure 3L, 3M). Follicle numbers were normalized to the area of the histological section, and each data point represented the average follicle number per area for each animal (*n* = 3 histological sections per animal) (Figure 4A–4H and Supplementary Figure 1A–1D). Overall, there was a decrease in total follicle number as a function of age for CON animals which is consistent with the gross histology (Figure 4A and Supplementary Table 1). For CON animals, there were significant decreases in primordial, transitional primordial, primary, transitional primary, secondary, and multilayer follicles between young and old animals (Figure 4B–4G). For CR animals, there were statistically significant decreases in primary follicles and secondary follicles between young and old animals (Figure 4D, 4F). Pairwise comparisons could not be made for antral follicles as there was no statistical significance for either of the main effects in the two-way ANOVA. Although there was a significant effect of age on follicle number across all follicle classes (except antral), there was no effect of diet on follicle number based on analysis of sources of variation using two-way ANOVA (Supplementary Table 1).

We performed an additional analysis of follicle number divided by total slide number per ovary, as a proxy for ovarian volume. As mentioned previously, we noted a large range of ovarian size across individual animals within an experimental group, as well as differences between groups, with ovarian mass significantly lower in old animals compared to young animals (*p* = 0.0016, Table 1 and Supplementary Table 2). Standardized follicle number was calculated by summing the follicle number per follicle class in each individual animal across all three tissue sections and dividing by the total number of histologic slides generated for that animal. Similar age-related declines in follicle number were observed when comparing these standardized follicle numbers (Supplementary Figure 2). No effect of diet on standardized follicle number was observed in the main effects analysis (Supplementary Table 3). Raw follicle number data is presented in Supplementary Table 4.

We next evaluated the distribution of follicles across the different developmental classes for each of the four experimental groups (Figure 4I–4L). The follicle class distribution for young CON, young CR, and old CR animals was grossly similar, with the vast majority being primordial or transitional primordial follicles (Figure 4I, 4K, 4L). By contrast, the follicle distribution in old CON animals was substantially different, with less than half of follicles classified as primordial or transitional primordial (Figure 4J). When compared to young animals, the follicle class distribution for old control animals is significantly different (*X*^2^ (6, *N* = 467) = 234.6, *p* < 0.0001). By comparison, there was no statistically significant difference in follicle class distribution between old calorie-restricted animals and young animals (*X*^2^ (6, *N* = 336) = 12.54, *p* = 0.0509). We then plotted the follicle class distribution on the level of individual animals, which further demonstrates a similar follicle class distribution across the young CON, young CR, and old CR groups and confirms that these patterns are not due to particular animals (Figure 4M). Notably, this analysis revealed that the old CON animals appear quite distinct from the others given that 3/8 animals had no follicles and one had only transitional primary follicles in the analyzed histological sections (Figure 4M). Overall, these data suggest that although CR does not prevent the overall age-dependent loss in follicles, it does maintain a distribution of follicles across developmental classes that is more similar to young animals, with a similar proportion of primordial and transitional primordial follicles observed between old CR animals and young animals irrespective of CON or CR status (Figure 4I–4M).

### Menstrual cycle status is associated with follicle number

**Figure 5:**
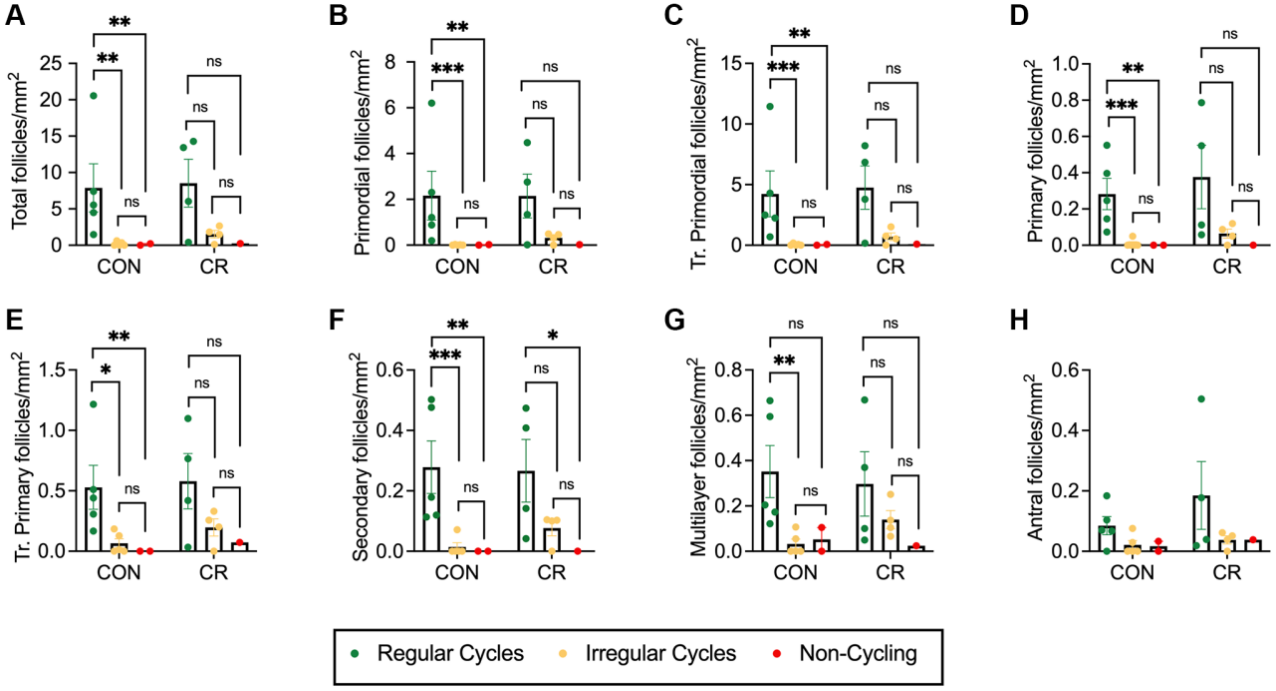
**Follicle number per area as a function of reproductive status.** Bar graphs showing follicle number per area for each normal follicle morphology class: (**A**) total number of follicles, (**B**) primordial, (**C**) transitional primordial, (**D**) primary, (**E**) transitional primary, (**F**) secondary, (**G**) multilayer, and (**H**) antral. Follicle number per area is plotted as untransformed data. Data presented as mean ± SEM. Green circles represent animals with regular cycles, yellow circles represent animals with irregular cycles, and red circles represent non-cycling animals. Statistics were calculated on log-transformed data with a two-way ANOVA. Tukey’s post hoc test was applied when one or both of the main effects were statistically significant. ^*^p ≤ 0.05, ^**^p ≤ 0.01, ^***^p ≤ 0.001. *P*-values for sources of variation of two-way ANOVA can be found in Supplementary Table 5.

In our study, the cohort of old animals included one animal with regular cycles (*n* = 1 CON), nine with irregular cycles (*n* = 5 CON, *n* = 4 CR), and three non-cycling animals (*n* = 2 CON, *n* = 1 CR). We, therefore, performed an additional analysis of how follicle number per area is affected by diet and menstrual cyclicity (Figure 5 and Supplementary Figure 3). For all follicle classes, there was a clear pattern of decreasing follicle number per area with increased menstrual cycle irregularity, with the lowest numbers of follicles seen in non-cycling animals for both CON and CR animals (Figure 5A–5H and Supplementary Figure 1E–1H). This pattern was also seen for standardized follicle number (Supplementary Figure 3). Statistically significant decreases in follicle number per area of irregularly cycling or non-cycling control animals compared to regularly cycling control animals were seen for all follicle classes, except antral follicles (Figure 5). The two-way ANOVA revealed that there was no statistically significant interaction between the effects of diet and menstrual cyclicity for total follicle number per area as well as follicle number per area for all normal and abnormal follicle classes (Supplementary Table 5). Simple main effects analysis showed that diet did not have a significant effect on total follicle number per area as well as follicle number per area for all normal and abnormal follicle classes (Supplementary Table 5). However, simple main effects analysis did show that menstrual cyclicity had a significant effect on the total follicle number per area, primordial follicle number per area, transitional primordial follicle number per area, primary follicle number per area, transitional primary follicle number per area, secondary follicle number per area, multilayer follicle number per area, multi-oocytic follicle number per area and atretic antral follicle number per area (Supplementary Table 5). Similar results in the main effects analysis were seen when looking at standardized follicle counts (Supplementary Table 6).

### Caloric restriction maintains the primordial follicle pool in a subpopulation of animals with irregular cycles

**Figure 6:**
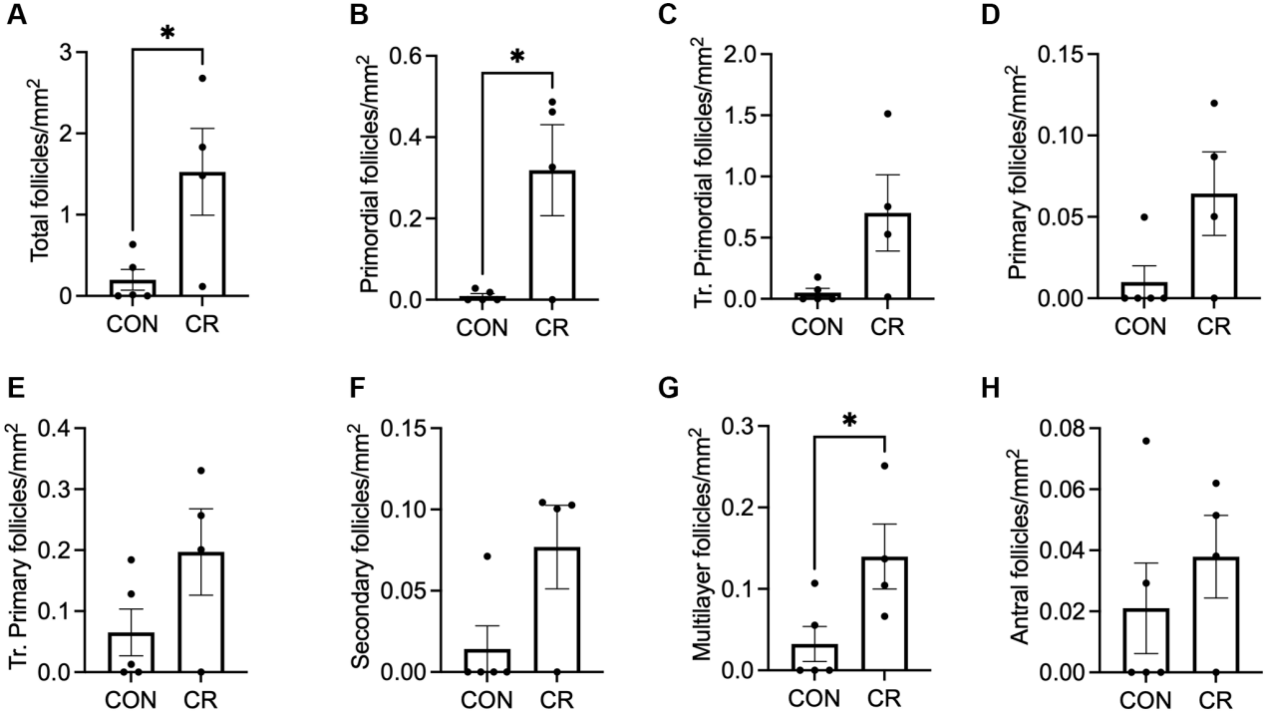
**Follicle number per area as a function of diet in old animals with irregular cycles.** Bar graphs of follicle number of normal morphology per ovarian section area are shown for the following classes: (**A**) total number of follicles, (**B**) primordial, (**C**) transitional primordial, (**D**) primary, (**E**) transitional primary, (**F**) secondary, (**G**) multilayer, and (**H**) antral follicles. Data are presented as mean ± SEM. Statistics were calculated using an unpaired *t*-test. ^*^p ≤ 0.05.

Previous studies have shown that the timing of CR interventions can modulate their impact [31, 32]. Although many studies focus on early (pre- or peri-pubertal) intervention [33–35], those aimed at extending the fertile window are likely to be to be more clinically relevant if initiated in later adulthood, when fertility is beginning to decline. Given the physiologic differences between aged animals with and without menstrual cycles, we performed a more focused analysis on the subgroup of irregularly cycling animals to determine the effect of CR in this clinically relevant population (Figure 6 and Supplementary Figure 1I–1L). Despite the lack of statistically significant effect of diet on follicle number in the cohort of all aged animals, it was physiologically reasonable to perform this subgroup analysis, given the significant physiological differences between cycling and non-cycling animals, and the statistically significant effect of menstrual cyclicity on follicle number. In the subgroup of irregularly cycling old animals, CR animals had significantly higher total follicle numbers per area than control (Figure 6A) which was primarily attributed to the significant increase in the number of primordial and multilayer follicles per area (Figure 6B, 6G), while other follicle classes did not differ (Figure 6C–6F, 6H). Despite the small follicle numbers in the abnormal follicle groups, which limits the power of the study, a significant increase in the secondary AMF follicle number per area in CR females was observed (Supplementary Figure 1J). However, a similar trend toward increasing follicle number per area with CR was seen for multilayer AMF follicles and atretic antral follicles (Supplementary Figure 1K, 1L). There was no effect of diet on multi-oocytic follicle number per area (Supplementary Figure 1I). Standardized follicle number for total follicles, follicles with normal morphologies, and follicles with abnormal morphologies are presented in Supplementary Figure 4. There was a statistically significant increase in only primordial follicle number with CR using this analysis (Supplementary Figure 4B), while total follicle number and other follicle classes did not differ (Supplementary Figure 4A, 4C–4L). Thus, a CR intervention later in life when an individual is experiencing irregular menstrual cyclicity may have a positive impact by preserving the primordial follicle pool and thereby potentially prolonging reproductive function.

### Age-related ovarian fibrosis is attenuated by CR

**Figure 7:**
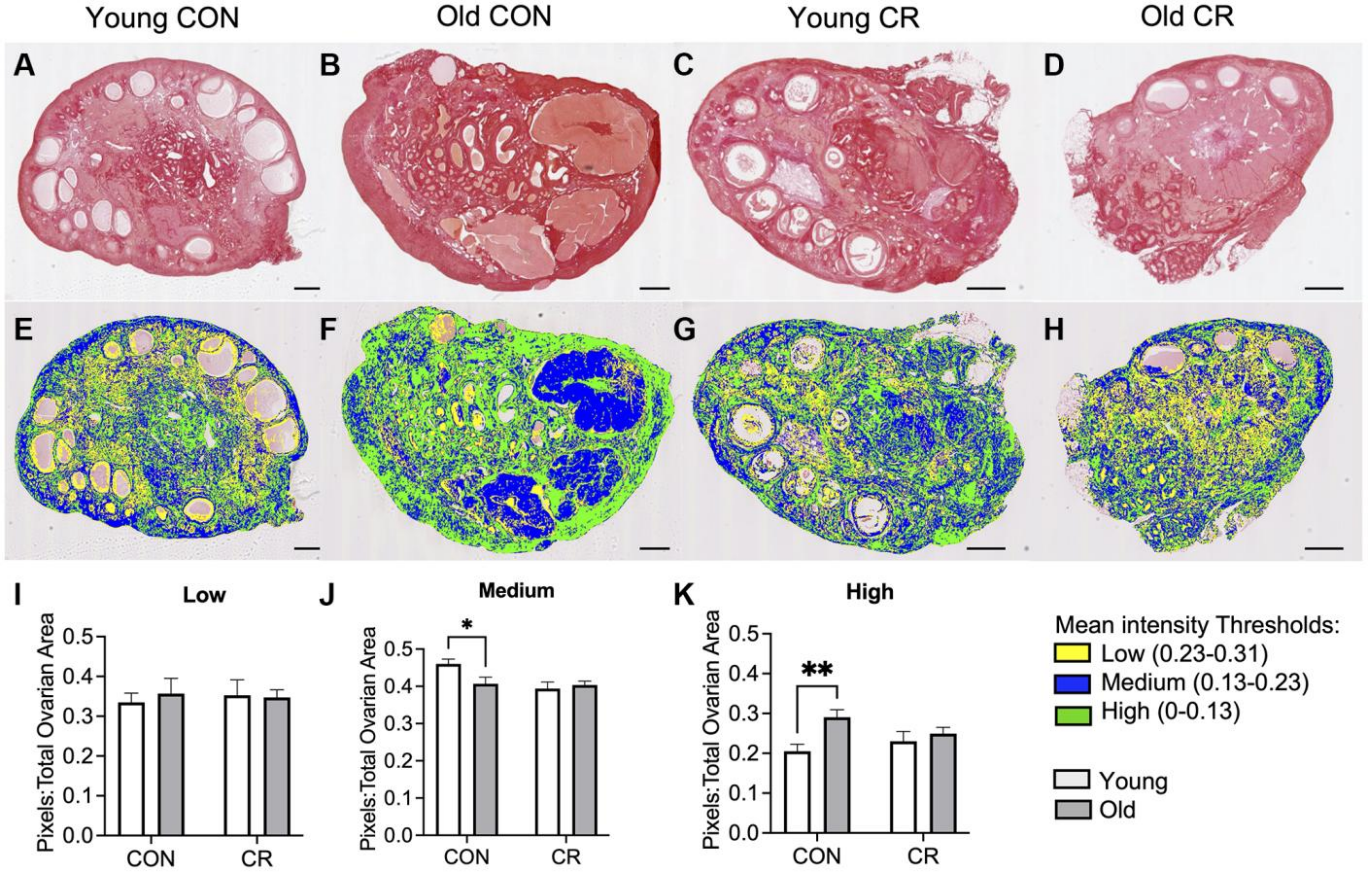
**Assessment of collagen I and III in histologic ovarian sections using Picrosirius red (PSR).** Representative images of PSR stained ovarian tissue sections for (**A**) young control, (**B**) old control, (**C**) young calorie-restricted (CR), and (**D**) old calorie-restricted (CR). Corresponding representative images of the quantitative histology algorithm showing intensity of PSR staining, where low intensity pixels are shown in yellow, medium intensity pixels are shown in blue, and high intensity are shown in green for (**E**) young control, (**F**) old control, (**G**) young calorie-restricted (CR), and (**H**) old calorie-restricted (CR). Bar graphs showing quantification of (**I**) low intensity, (**J**) medium intensity, and (**K**) high intensity pixels. An *N* = 3 images per animal and *N* = 4–8 animals per experimental group were analyzed. Data are presented as mean ± SEM. Statistics were calculated using a two-way ANOVA with Sidak’s multiple comparison test. ^*^p ≤ 0.05. ^**^p 0.01. The scale bar is 1 mm.

**Figure 8:**
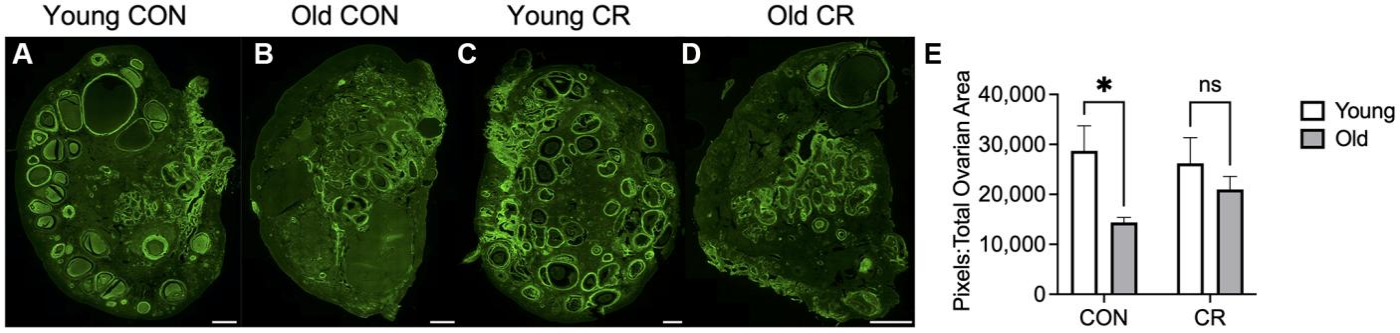
**Hyaluronic acid binding protein localization in ovarian tissue sections.** Representative fluorescent microscopy images of hyaluronic acid binding protein (HABP) showing localization of hyaluronic acid in ovarian tissue sections in (**A**) young control, (**B**) old control, (**C**) young calorie-restricted (CR), and (**D**) old calorie-restricted (CR) animals. Quantification of the positive pixels per area is shown in (**E**) with *N* = 3 images per animal and *N* = 4–8 animals per experimental group. Data are presented as mean ± SEM. Statistics were calculated using a two-way ANOVA with Sidak’s multiple comparison test. ^*^p ≤ 0.05. The scale bar is 1 mm.

Distinct changes in the ovarian extracellular matrix occur with advanced reproductive age, including an increase in collagen and a decrease in hyaluronic acid which are associated with increased tissue stiffness [4, 36, 37]. To investigate whether CR had an impact on age-related changes in ovarian extracellular matrix, we evaluated collagen and hyaluronic acid (HA). We performed Picrosirius Red staining which is a histologic stain that can be used to detect collagen I and III fibers, and we used quantitative digital pathology to quantify pixels of high, medium, and low intensity in ovarian sections across experimental groups, based on our prior work (Figure 7) [38]. As expected with age, there was an increase in collagen content in old CON ovaries (Figure 7B, 7F) relative to young CON ovaries (Figure 7A, 7E) as evidenced qualitatively by the increase in red staining and quantitatively by the age-dependent increase high intensity pixels (Figure 7D, 7H, 7K) with no CR effects in young animals (Figure 7C, 7G). However, this age-dependent increase in ovarian collagen was abrogated in ovaries from old CR animals (Figure 7K). With increased age, there was a decrease in medium intensity pixels in control animals, suggesting a change in the collagen matrix (Figure 7J). No statistically significant differences between groups were seen for low intensity pixels (Figure 7I). In the aging mouse and human ovary, the increase in collagen is accompanied by a decrease in hyaluronic acid [4, 36, 39]. Using a fluorescent hyaluronic acid binding protein (HABP) assay to localize hyaluronic acid within ovarian sections, we noted that the loss of hyaluronic acid with age is also conserved in the rhesus macaque in CON samples (Figure 8A, 8B). However, this age-related difference was not significant in CR animals (Figure 8C–8E). Together these results suggest that CR may attenuate age-related fibrosis in the ovary.

## DISCUSSION

In this study, we provide evidence that short-term moderate CR confers a beneficial effect on reproductive longevity in the NHP model but may be dependent on the timing of when CR is administered with respect to the reproductive lifespan of the animal. CR preserved a more youthful distribution of follicles among aged animals through preservation of a higher proportion of primordial follicles, while not necessarily attenuating the overall declining number of follicles. We established that CR maintains the number of primordial follicles in irregularly cycling animals, supporting the notion of CR timing as a modulator of ovarian impact. Finally, we found that CR attenuates age-related changes in collagen and hyaluronic acid matrices which are both implicated in ovarian fibrosis. These main findings suggest that CR may improve reproductive longevity, with timing as a critical factor. Moreover, our study established a novel follicle-class categorization method for the NHP model which is critical for future studies that rely on this important ovarian endpoint. This study also defines the age-related decline in follicle number both in cycling and non-cycling animals and provides a quantitative metric of reproductive longevity in the NHP model.

There is a large degree of variation in the existing literature with respect to the age of onset, duration, and degree of the CR intervention. The data demonstrate that the timing of the CR intervention matters and may be species-specific. Early onset CR in rodents increased longevity compared to adult-onset CR [33, 35]. Yet, CR implemented in NHP juveniles does not seem to improve survival or longevity [14]. Hence, while CR effectively delays the effects of aging in NHP, the extent of CR benefits relates to the age of onset of the CR intervention [20]. Our study supports previous studies in NHP, that CR in young cohorts may not necessarily improve reproductive longevity and supports the notion that the age of onset of CR matters. Moreover, we propose a novel notion that menstrual status during ovarian aging, rather than age alone, may play an important role in the impact of CR on the declining ovarian reserve.

In our study, we investigated a short-term (3 year) period of CR. Studies in rodents have reported long-term CR interventions, with some studies even reporting life-long moderate CR [40]. Prior studies in rhesus macaques carried out by the National Institute on Aging (NIA) and Wisconsin National Primate Research Center (WNPRC) have reported decades-long CR interventions, with mixed effects [14, 20]. Such long-term CR is likely not sustainable or realistic in NHP or humans, and some have raised concerns about the safety of such long-term interventions, with regards to bone mineral density, impaired immune function, and nutrient deficiencies [41]. Intermittent fasting may offer an more acceptable, feasible alternative to long-term caloric restriction and studies have shown beneficial effect on ovarian aging parameters in mice [42]. The impact of intermittent fasting in primates remains to be determined. Finally, the degree of CR is an important consideration. CR at a higher level of 40% delayed ovulation in macaques through an endocrine effect caused by a negative energy balance [43]. Our study’s use of 30% CR suggests a similar level of diet restriction impacts ovarian mechanisms in NHP. Further research is needed to determine the minimum degree of CR that confers a benefit in NHP.

The exact mechanisms by which CR exerts its anti-aging effects and promotes ovarian longevity remain unknown. However, the beneficial effects of CR are likely multi-factorial, including positive effects on insulin sensitivity, inflammation, energy metabolism, oxidative stress, autophagy, and the neuroendocrine axis [44, 45]. Multiple molecular pathways have been hypothesized to be involved, including mTOR and the related SIRT pathways [46]. Indeed, other studies have analyzed pharmacologic interventions such as rapamycin, which maintain the ovarian reserve through mTOR inhibition [47] and sirtuin-activating compounds [48], which reduced oxidative stress through the sirtuin pathway. Our results show that CR attenuates the ovarian age-related fibrotic process. Metformin, a common anti-diabetic medication, has also been shown to decrease ovarian fibrosis [49, 50]. Studies have shown that the reduction of ovarian fibrosis through anti-fibrotic drugs has been shown to extend female reproductive lifespan via an increase in ovulation capacity in a mouse model [51]. Therefore, because CR attenuates fibrosis in the NHP model, it may further suggest CR as a tool to extend reproductive lifespan, as the fibrotic process is directly linked to ovulation capacity. Further studies identifying the mechanisms of CR underlying the regulation of ovarian lifespan are needed so that therapeutic approaches to target these pathways may be developed to maintain ovarian reserve.

Limitations of this study include the retrospective nature of analysis and non-uniform group sample sizes. Rhesus macaques, like humans, are genetically heterogeneous and may therefore display varied phenotypes in response to the same environmental stimuli [20]. For example, animals progressed through their reproductive lifespans spontaneously, so number of animals regularly-cycling, irregularly cycling, and non-cycling was not consistent between experimental groups. Finally, our study analyzed the impact of only one level of CR (30% caloric restriction) and does not address the impact of different degrees of CR, which may be an important modulator of its effect on ovarian longevity. Another limitation is that only a subset of histological sections was analyzed per animal. Use of sampling strategies (counting a subset of histological sections and applying a correction factor to account for uncounted tissue), is a common approach in the follicle counting literature and as long as same technique is applied uniformly to all experimental groups, results in internal validity [52].

In our study, although CR did not significantly increase the overall number of follicles, CR maintained a youthful proportion of follicle class, preserved primordial follicles in irregularly cycling animals, and attenuated ovarian fibrosis. The clinical implications are relevant, and our study provides evidence of CR as a possible intervention to attenuate the loss of ovarian function with aging. Through the NHP model, we provide findings that can be extended to humans more specifically than other model systems. The impact of the relationship between CR and other molecular pathways on ovarian aging remains to be determined, but may provide an important insight to the mechanism of CR as an intervention and support the use of CR mimetics. Optimal timing, duration, and degree of CR intervention needed to promote reproductive longevity in women remains to be determined by future studies.

## MATERIALS AND METHODS

### Animals

We acquired a subset of archived ovarian tissue samples from a prior study involving short-term moderate CR in the rhesus macaque in two age cohorts (Figure 1). In this study, a cohort of young and old (peri-menopausal) female rhesus macaques (*M. mulatta*) was selected under a protocol approved by the Institutional Animal Care and Use Committee (IACUC) of the Oregon National Primate Research Center. The animals were housed in individual cages with auditory, visual, and olfactory interaction with female conspecifics in a temperature-controlled environment (24°C) under a fixed 12L:12D photoperiod (lights on from 700 h to 1900 h) with *ad libitum* access to drinking water. Animals were cared for by the Oregon National Primate Research Center in accord with the National Research Council’s Guide for the Care and Use of Laboratory Animals [53], which included daily health checks to ensure normal behavior, food consumption, and waste production. Additionally, routine physical examinations, hematological studies, fecal parasite checks, tuberculin testing, and dental cleaning were performed periodically.

### Diet information

Animals in both the young and old cohorts were randomly assigned to control (CON) or caloric restriction (CR) diet. While the original CR studies in rodents performed by McCay et al. [8] use a 40% caloric restriction, when designing this study for non-human primates, a 30% reduction in calories was decided upon based on considerations of safety, compliance, and ability to perform the intervention in juvenile animals still in their growth phase when translation of McCay’s seminal work to a larger animal species. The experimental cohorts for this study included: (1) young/CON (young animals on the control diet; *N* = 4), (2) young/CR (young animals on the caloric restriction diet; *N* = 4), (3) old/CON (old animals on the control diet; *N* = 8), (4) old/CR (old animals on the caloric restriction diet; *N* = 5) (Figure 1 and Table 1). Animals in the control group were provided a daily allotment of food, adjusted for age and body weight. The average daily value was then divided into two meals. The CR diet was then reduced by 10% per month until a 30% CR was achieved as previously described [31, 32, 54, 55]. CR animals received 30% less food than age- and body weight-matched CON animals. Each animal received a measured portion of specially formulated biscuits (Cargill, Minneapolis, MN, USA) supplemented with daily fresh fruits or vegetables (10–40 cal) and was fed twice daily, at 0800 h and 1500 h. Biscuit composition was 15% protein, 5% fat, and 5% fiber, with a caloric content of ∼3.7 kcal/g. To avoid any deficiencies in essential nutrients in the CR animals, the diet was enriched with a vitamin/mineral mix that was 40% higher than the recommended allowance for rhesus macaques by the National Research Council [53]. These animals were calorie restricted but not malnourished. Animals were maintained on a control (CON) or a moderate CR diet for up to 3 years. Biochemical assays were performed periodically and with every new shipment to ensure diet content and quality [56, 57].

### Menstrual cyclicity analysis

We used hormone data from the Endocrine Services Laboratory Core, Oregon National Primate Research Center (Roche, Beaverton, OR, USA) to assign animals into groups of those who had regular cycles and those that did not. Steroid (estradiol (E2), progesterone (P4)) and gonadotropin hormones (follicle-stimulating hormone (FSH), luteinizing hormone (LH)) were retrospectively synchronized relative to the estimated mid-cycle LH peak (designated LH d0). This separates the follicular and luteal phases to permit analyses between comparable physiological states. Based on these data, animals were assigned to one of two groups: regular cyclers and irregular cyclers. Regular cyclers exhibited 3 cycles each demonstrating a mid-cycle E2 peak followed by luteal phase levels of P4 during the 90-day interval. Irregular cyclers typically exhibited at least one cycle within the 90-day interval with a mid-cycle E2 peak followed by basal levels of P4 indicating the absence of a luteal phase. The remaining two cycles within the 90-day interval were characterized by very low levels of E2 and P4 lacking cyclic rises, with no further observation of menses. Non-cycling animals displayed very low hormone activity, with no recorded menses noted and little to no change in both E2 and P4. After the 90-day sampling period (between 1–2 years on CR), menses records were reported every month until the completion of the experiment at the end of three years. Using the definition from Gilardi and colleagues [25], an animal was classified as an irregular cycler if she exhibited 6 consecutive months or more of irregular cycles, excluding the non-breeding season (June through August). Data on hormone levels and response to ovarian stimulation will be presented in an accompanying manuscript.

### Histological samples

At the end of the study period, animals were sacrificed, and ovaries were harvested. Ovaries were fixed in 4% paraformaldehyde (Sigma-Aldrich, St. Louis, MO, USA) for 24 hours at 4°C, 4% sucrose (Sigma-Aldrich, St. Louis, MO, USA) for 24 h at 4°C, and stored in 70% ethanol (DeCon Labs, Inc., King of Prussia, PA, USA) at 4°C for up to one week prior to dehydration and embedding in paraffin. The ovaries were serially sectioned at 5 μm thickness and mounted onto slides with two sections per slide. As it has been previously demonstrated that the number of primordial follicles did not differ between right and left ovaries in rhesus macaques only one ovary per animal was serially sectioned and used for histological analysis in this study [58]. These slides were used for downstream analyses as described below.

### Follicle classification and counting

A subset of histological sections (every 10th slide) was stained with hematoxylin and eosin (H&E) to visualize ovarian follicles and other tissue structures. Samples were deparaffinized in Citrosolv (Decon Laboratories, King of Prussia, PA, USA) and rehydrated in a series of graded ethanol baths (100%, 75%, 70%) and a standard H&E staining protocol was used. H&E-stained slides were imaged using the NanoZoomer Digital Pathology System HT-9600 (Hamamatsu, Hamamatsu City, Japan). Images were then accessed using NDP Viewer Software (Hamamatsu, Hamamatsu City, Japan). Follicle counting was performed in the NDP Viewer software under 40X magnification. To account for variation in ovary size and tissue architecture, three representative sections per animal were selected for analysis at one quarter (1), midway (2), and three quarters (3) through the ovary (Figure 3L). Each section was radially divided into 12 wedge-shaped segments to facilitate counting (Figure 3M). Within each segment, each follicle type was assigned a colored marker based on the classification system described below. Three individual counters counted a section at the same location in the ovary across all animals (e.g., section 1, 2, or 3, Figure 3L). To ensure rigor and reproducibility, for every 12 samples analyzed (top, middle, and bottom section from an animal in each of the four experimental groups), a calibration count was performed as follows. For each section 1, 2, or 3, an individual counter blinded to the original count re-analyzed one wedge-shaped segment from the section. Thus, three wedges per 12 histological samples were re-analyzed. *A priori* it was established that total follicle counts must be within 5% error, or the entire histological section was reanalyzed by the second counter, and counts were then averaged. No histological sections met the requirement for reanalysis by a second counter. Follicle counts were totaled and reported by follicle class.

The protocol for classifying follicles for counting purposes was adapted for NHP based on categories established during discussions held at the 2021 Ovarian Anatomy Nomenclature Workshop led by the National Institutes of Health [59]. For morphologically normal follicles, a primordial follicle was defined as having an oocyte surrounded by a complete or incomplete layer of squamous granulosa cells (Figure 3A). A transitional primordial follicle was one that has an oocyte surrounded by a complete or incomplete layer of both squamous and cuboidal granulosa cells (Figure 3B). A primary follicle contained an oocyte surrounded by only cuboidal granulosa cells which formed a compact and complete (or nearly complete) layer (Figure 3C). A zona pellucida between the oocyte and granulosa cells was also visible at this stage. A transitional primary follicle consisted of an oocyte surrounded by more than 1, but fewer than 2 complete layer(s) of cuboidal granulosa cells (Figure 3D). Follicles were classified as secondary when the oocyte was surrounded by 2–3 layers of cuboidal granulosa cells and multilayer when surrounded by 3+ such layers (Figure 3E, 3F). Classification was based on the maximum number of layers on any given side of the follicle. Antral follicles were large and contained a fluid-filled antral cavity which spanned multiple sections (Figure 3G). An outer layer of theca cells was typically visible in follicles at the secondary stage and beyond and was distinctly formed in antral follicles. To avoid double counting follicles, antral follicles were not counted in the middle section of each animal. Otherwise, follicles were classified regardless of the presence of an oocyte nucleus, as the sections analyzed were sufficiently far apart.

In addition to morphologically normal follicles, we also classified and quantified follicles with abnormal morphological features. A multi-oocytic follicle contained two or more oocytes within one follicle structure enclosed by a basement membrane (Figure 3H). A secondary or multilayer follicle with abnormal morphological features (secondary AMF, multilayer AMF) contained structural abnormalities in the oocyte and/or granulosa cells, such as the presence of vacuoles in more than 50% of the oocyte (Figure 3I, 3J). Other criteria leading to an AMF classification included a damaged, misshapen, or degenerating oocyte, nucleus, and/or granulosa cells, or the severe shrinkage of the oocyte and/or granulosa cells away from the stroma in a section where most follicles were clearly identifiable suggesting that the phenotype was not a fixation artifact. Atretic antral follicles were defined as those with significant numbers of granulosa cells which had separated from the mural layer and localized within the antral cavity. Atretic antral follicles typically also exhibited other AMF features in the nucleus, oocyte, and/or granulosa cells (Figure 3K). Follicle number per area was calculated by dividing the follicle number by the area of the histologic section, which was measured using Fiji (ImageJ, National Institutes of Health, Bethesda, MD). Standardized follicle number was calculated by summing the follicle number per follicle class in each individual animal across all three tissue sections and normalizing to the total number of histologic slides generated for that animal. To generate the pie charts in Figure 4I–4L, raw follicle number from each follicle class was summed for all animals in a given experimental group and these sums were plotted to show the distribution of follicles from each class. As there are different numbers of animals in each experimental group and different numbers of follicles counted for each animal, each pie chart depicts the follicle distribution for a different number of animals/follicles (for: (I) *N* = 4 animals, *n* = 3315 follicles); (J) *N* = 5 animals, *n* = 467 follicles); (K) *N* = 4 animals, *n* = 3448 follicles; and (L) *N* = 5 animals, *n* = 336 follicles). Animals without follicles were not included in pie charts.

### Picrosirius red staining and collagen quantification

For assessment of collagen I and collagen III, ovarian sections (*N* = 4–8 animals/group, *N* = 6 sections/ovary) were stained with Picrosirius Red (PSR) according to established protocols [36]. Briefly, tissue sections were deparaffinized in Citrosolv (Decon Laboratories) and then rehydrated in a series of graded ethanol baths (100%, 70%, and 30%). Slides were submerged in a PSR staining solution, which was prepared by dissolving Sirius Red F3B (Direct Red 80, Color Index 35780, Sigma-Aldrich, St. Louis, MO, USA) in a 1.3% saturated aqueous picric acid solution (Sigma-Aldrich, St. Louis, MO, USA) at 0.1% w/v. Slides were immersed in the PSR staining solution for 30 minutes at room temperature, then destained with acidified water. Samples were then rapidly dehydrated in 100% ethanol, cleared in xylene, and mounted with synthetic mounting media. Quantitative digital microscopy was used to analyze the collagen content in the PSR-stained tissue sections as previously described [38]. Stained slides were imaged using the NanoZoomer Digital Pathology System HT-9600 (Hamamatsu, Hamamatsu City, Japan). Images were then imported into Visiopharm software (Visiopharm, Hoersholm, Denmark) and analyzed using the Image Analysis Module. Three slides (top, middle, and bottom) with two adjacent sections were analyzed for each animal. Regions of interest (ROIs) were drawn around each ovarian tissue section and assigned to “right” or “left” ovary. Then, these images were converted into grayscale values using the Chromaticity Green feature band. The software labeled each pixel as low, medium, or high degree of staining based on thresholds used in our prior work [38]. Images were processed in batch mode using this algorithm. The area of pixels with low, medium, and high staining intensity was calculated per total ovarian area and averaged between left and right ovarian sections.

### Hyaluronic acid binding protein assay and hyaluronic acid quantification

A hyaluronic acid binding protein (HABP) assay was performed to localize hyaluronic acid within histological sections of ovarian tissue as previously described [39, 60]. In brief, tissue sections were deparaffinized in Citrosolv (Decon Laboratories) and then rehydrated in a series of graded ethanol baths (100%, 95%, and 95%). Slides were incubating in running reverse-osmosis water for 1 minute and washed in 1X phosphate buffered saline (PBS) with gentle rocking for 10 minutes. An Avidin/Biotin Blocking Kit (Vector Laboratories, Burlingame, CA, USA) was used according to kit instructions to block endogenous avidin and biotin within the tissue sections. Avidin was applied for 15 minutes at room temperature. Unbound avidin was rinsed off using 1X PBS, then biotin was applied for 15 minutes at room temperature. Unbound biotin was rinsed off using 1X PBS and slides were subsequently incubated in normal goat serum (Fisher Scientific, Waltham, MA, USA) for 20 minutes. Next, 1 mg/mL hyaluronidase (Sigma-Aldrich, St. Louis, MO, USA) in saline solution was applied to negative control tissue sections and saline alone was applied to experimental sections. Tissue sections were incubated in a humid chamber for 1 hour at 37°C. After rinsing in 1X PBS, biotinylated HABP (Calbiochem, San Diego, CA, USA) diluted in normal goat serum was applied to all sections and incubated for 1 hour at room temperature. Slides were again washed in 1X PBS. To amplify signal, slides were incubated in ABC reagent (Vector Laboratories, Burlingame, CA, USA) for 30 minutes at room temperature, followed by TSA Plus Fluorescein System (Akoya Biosciences, Marlborough, MA, USA) at a 1:400 dilution for 5 minutes at room temperature. All incubations were performed within a humified chamber. Samples were mounted in Vectashield HardSet Antifade Mounting Medium with DAPI (4′,6-diamidino-2-phenylindole; Vector Laboratories, Burlingame, CA, USA) to counterstain cell nuclei. Sections were processed in batches containing one slide from each animal. The HABP assay was performed on three replicates from each animal. Entire ovarian tissue sections were scanned at 10X using a Leica DM6B Fluorescent Microscope (Leica Biosytems, Deer Park, IL, USA). The imaging settings were kept constant for all samples after determining the threshold. As a control, the second tissue section on the slide was treated with hyaluronidase and we confirmed that the hyaluronidase-treated samples did not show a positive signal and used this to help set the threshold. HABP staining intensity analysis was performed using Fiji (ImageJ). Hyaluronic acid staining is reported as a ratio of HABP intensity per area in the total ovarian section. The background was determined using the hyaluronidase-treated sections, and this value was subtracted from the hyaluronic acid positive sections.

### Statistical analysis

Data were analyzed using Prism Software version 9.3.1 (GraphPad, La Jolla, CA, USA) and R version 4.4.2. Descriptive statistics expressed as the mean ± standard error of means (SEM). Visual inspection demonstrated follicle number per area and standardized follicle number data to be skewed and contained clusters of zeros. Therefore, the original data set was log transformed. For outcomes with positive values, the natural logarithm was taken directly. For outcomes with zero values, a small constant (half of the smallest positive outcome value) was added before applying the logarithm to avoid undefined values. We used unpaired *t*-tests to determine significance between two groups and one-way analysis of variance (ANOVA) with a Tukey’s post hoc test to determine significance between three or more groups. To evaluate the effect of two independent variables (diet and age or menstrual cyclicity) on follicle number, two-way ANOVA was used. Levene’s test for homogeneity of variances was performed for all two-way ANOVA analyses; all models met the homogeneity assumption except for one (multilayer follicle number per area), which showed borderline heteroscedasticity (*p* = 0.042). Tukey’s post hoc test was applied when one or both of the main effects were statistically significant. To compare the follicle distribution pie charts in Figure 4, Chi-square test was used to compare the observed distribution for each experimental group against the average distribution of young control and young calorie-restricted animals. For all statistical analyses, significance was set at unadjusted or adjusted *p* < 0.05. In all figures, data are plotted untransformed and statistical tests are performed on log transformed data. P-values for main effects and interaction testing from two-way ANOVAs are shown in Supplementary Tables 1, 3, 5, and 6. Statistically significant pairwise comparisons are shown visually in the figures with asterisks signifying level of significance.

## Supplementary Materials

Supplementary Figures

Supplementary Tables
