# Supplementary material for: Short-term moderate caloric restriction in the rhesus macaque attenuates markers of ovarian aging in select populations
Source: Aging (Albany NY). 2025 May 20;17(5):1164–89. doi: 10.18632/aging.206253 (PMC12151502; doi:10.18632/aging.206253)
Supplement: Supplementary Figures [file aging-17-206253-s001.pdf]

SUPPLEMENTARY FIGURES

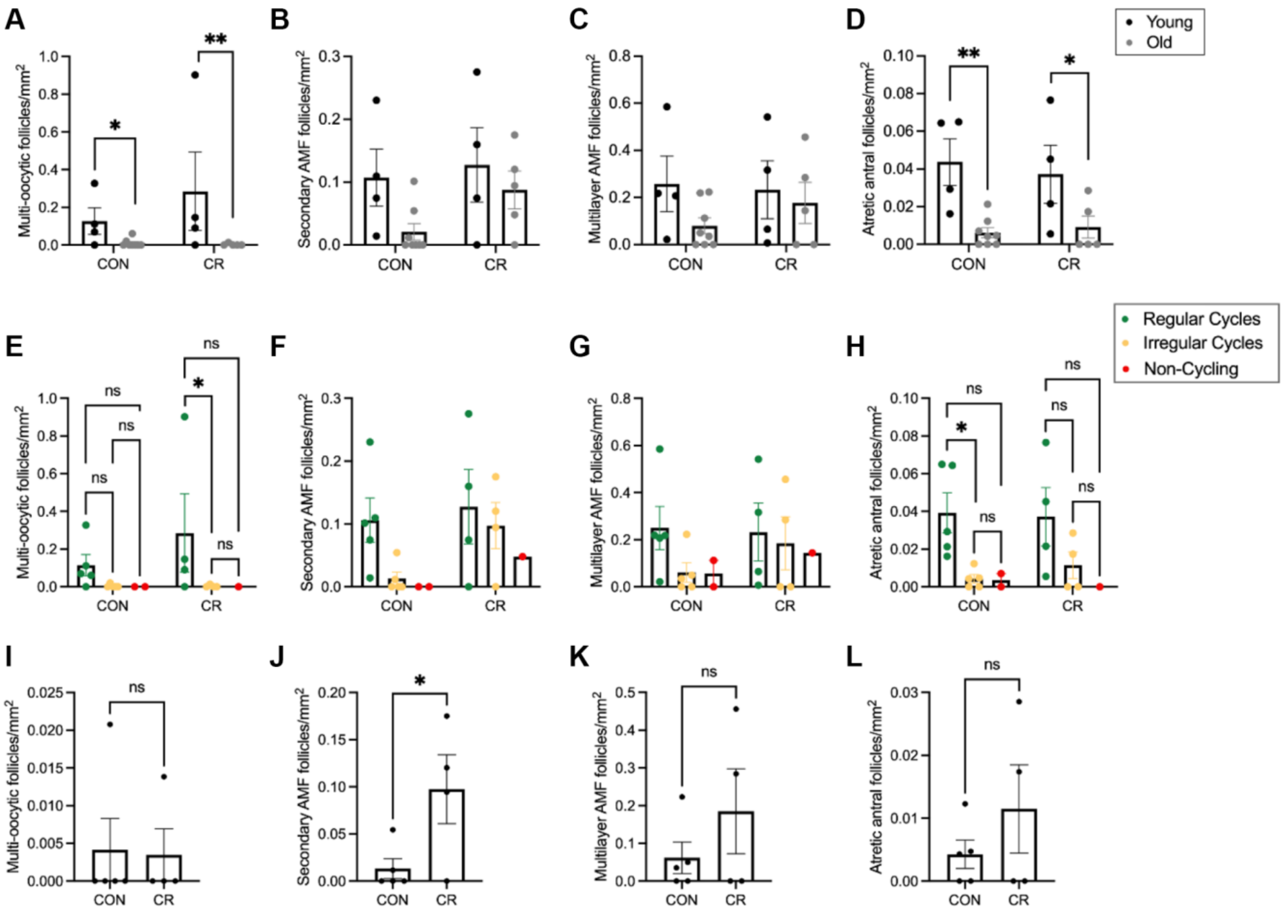

Supplementary Figure 1. Follicle number for abnormal follicle morphologies per ovarian section area. Bar graphs comparing the effect of age and diet on follicle number per ovarian section area are shown for each abnormal follicle morphology class including: (**A**) multi-oocytic, (**B**) secondary with abnormal morphologic features (AMF), (**C**) multilayer with AMF, and (**D**) atretic antral follicles. Bar graphs comparing the effect of reproductive status and diet on follicle number per ovarian section area for each abnormal follicle morphology class including: (**E**) multi-oocytic, (**F**) secondary with AMF, (**G**) multilayer with AMF, and (**H**) atretic antral follicles. Bar graphs comparing the effect of diet on follicle number per ovarian section area for each abnormal follicle morphology class are shown in old animals with irregular cycles including: (**I**) multi-oocytic, (**J**) secondary with AMF, (**K**) multilayer with AMF, and (**L**) atretic antral follicles. Data are presented as mean ± SEM. In (**A**–**D**), black circles represent young animals and gray circles represent old animals. In (**E**–**H**), green circles represent animals with regular cycles, yellow circles represent animals with irregular cycles, and red circles represent non-cycling animals. Untransformed data are plotted. Statistics were calculated using a two-way ANOVA with Tukey's multiple comparison test (if appropriate) on log-transformed data for (**A**–**H**) and an unpaired *t*-test on untransformed data (**I**–**L**). *P*-values for sources of variation of two-way ANOVA can be found in Supplementary Tables 1 and 5. $^{*}p \leq 0.05$ and $^{**}p \leq 0.01$.

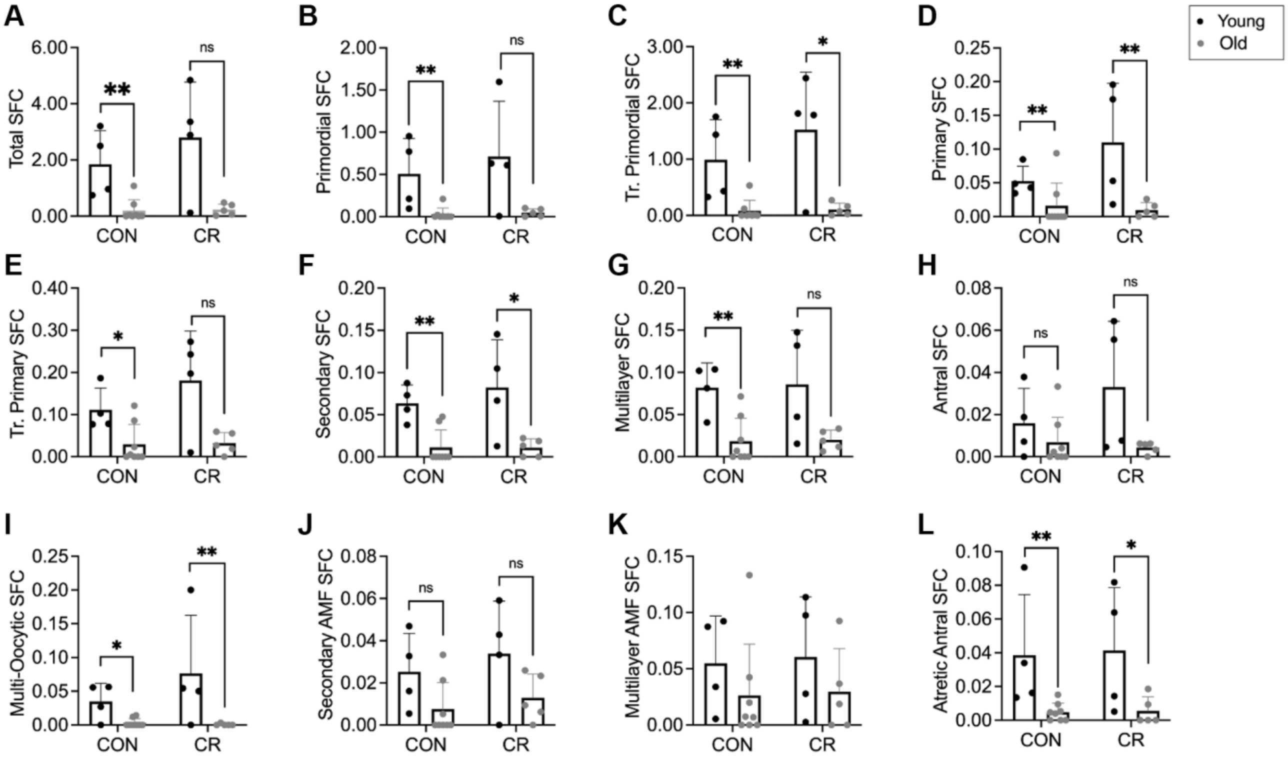

Supplementary Figure 2. Standardized follicle number as a function of age and diet. Standardized follicle number was calculated by summing the follicle number per class in each individual animal and dividing by the number of histologic slides generated for that animal. Bar graphs highlighting standardized follicle number for each normal and abnormal follicle morphology class are shown including: (**A**) total number of follicles, (**B**) primordial, (**C**) transitional primordial, (**D**) primary, (**E**) transitional primary, (**F**) secondary, (**G**) multilayer, (**H**) antral, (**I**) multi-oocytic, (**J**) secondary with abnormal morphologic features (AMF) (**K**) multilayer with AMF, and (**L**) atretic antral follicles. Data are presented as mean ± SEM. Black circles indicate young animals and grey circles indicate old animals. Untransformed data are plotted. Statistics were calculated using a two-way ANOVA with Tukey's multiple comparison test (if appropriate) on log-transformed data. *P*-values for sources of variation of two-way ANOVA can be found in Supplementary Table 3. $^{*}p \leq 0.05$, $^{**}p \leq 0.01$.

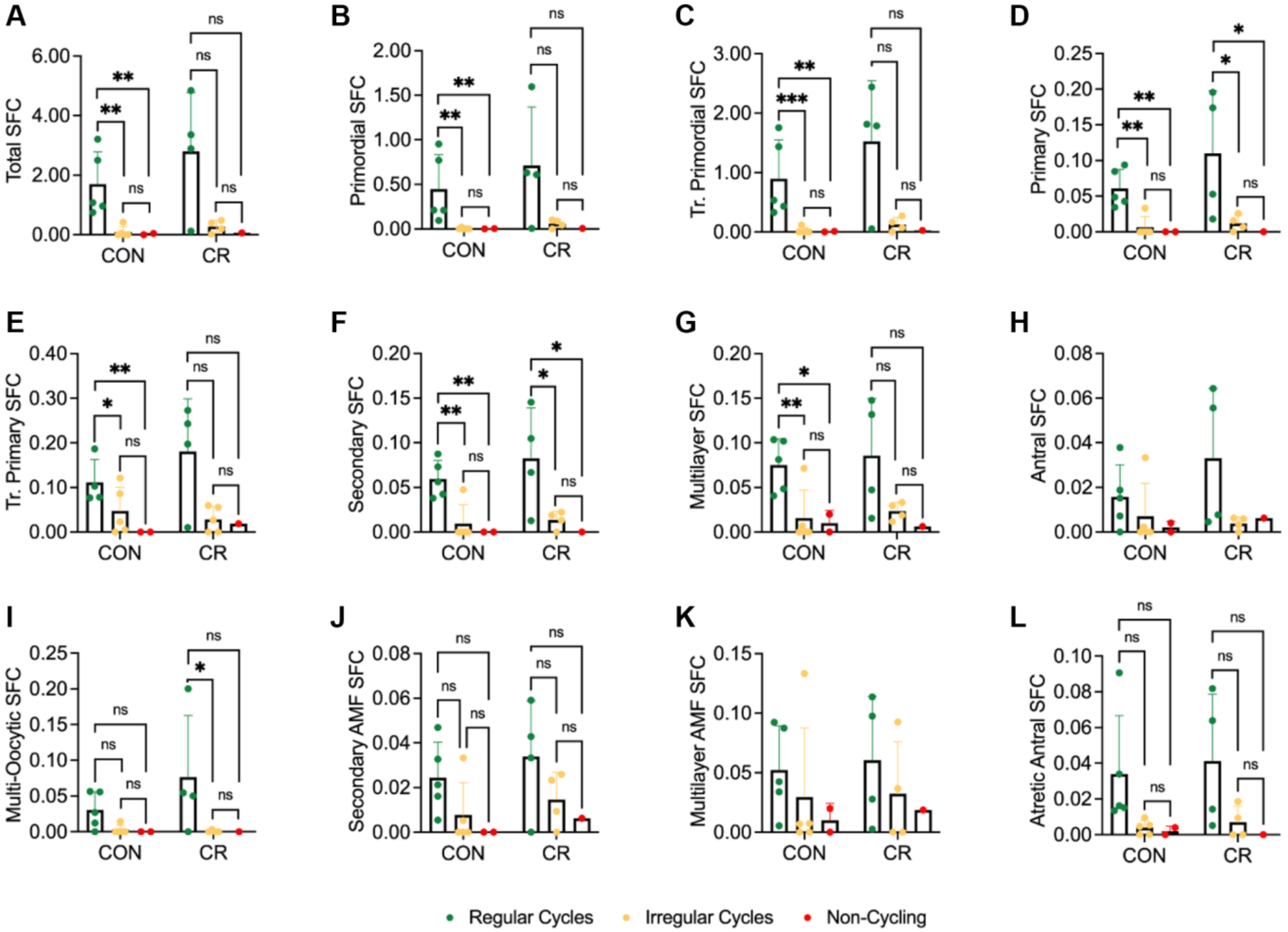

Supplementary Figure 3. Standardized follicle number as a function of reproductive status and diet. Standardized follicle number was calculated by summing the follicle number per class in each individual animal and dividing by the number of histologic slides generated for that animal. Bar graphs highlighting standardized follicle count for each normal and abnormal follicle morphology class are shown including: (**A**) total number of follicles, (**B**) primordial, (**C**) transitional primordial, (**D**) primary, (**E**) transitional primary, (**F**) secondary, (**G**) multilayer, (**H**) antral, (**I**) multi-oocytic, (**J**) secondary with abnormal morphologic features (AMF) (**K**) multilayer with AMF, and (**L**) atretic antral follicles. Data are presented as mean ± SEM. Green circles represent animals with regular cycles, yellow circles represent animals with irregular cycles, and red circles represent non-cycling animals. Untransformed data are plotted. Statistics were calculated with a two-way ANOVA with Tukey's multiple comparison test (if appropriate) on log-transformed data. $^{*}p \leq 0.05$, $^{**}p \leq 0.01$, $^{***}p \leq 0.001$. *P*-values for sources of variation of two-way ANOVA can be found in Supplementary Table 5.

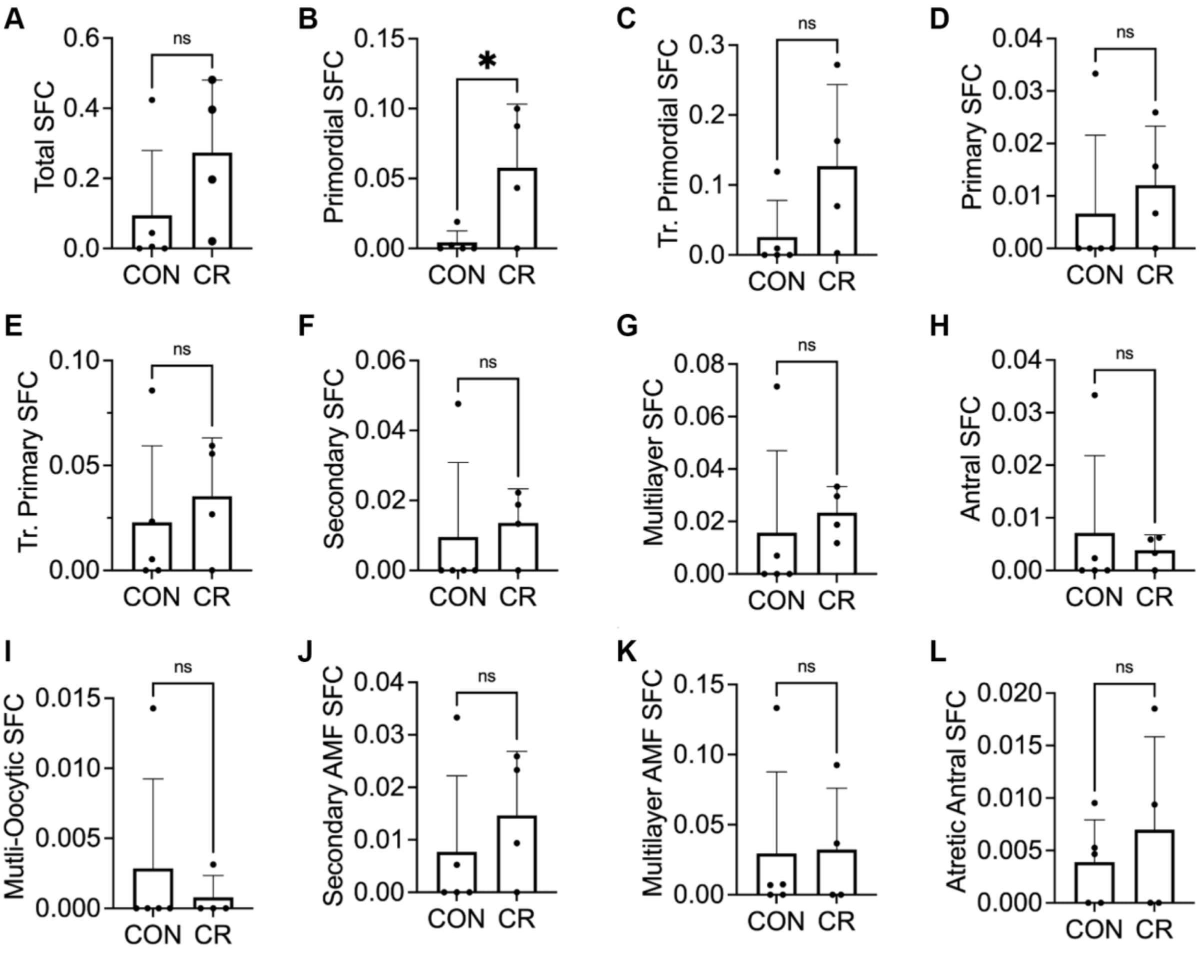

Supplementary Figure 4. Standardized follicle number as a function of diet in old animals with irregular cycles. Standardized follicle number was calculated by summing the follicle number per class in each individual animal and dividing by the number of histologic slides generated for that animal. Bar graphs highlighting standardized follicle number for each follicle morphology class are shown including: (**A**) total number of follicles, (**B**) primordial, (**C**) transitional primordial, (**D**) primary, (**E**) transitional primary, (**F**) secondary, (**G**) multilayer, (**H**) antral, (**I**) multi-oocytic, (**J**) secondary with abnormal morphologic features (AMF) (**K**) multilayer with AMF, and (**L**) atretic antral follicles. Data are presented as mean ± SEM. Statistics were calculated using an unpaired *t*-test. $^{*}p \le 0.05$.
