# Supplementary material for: Short-term moderate caloric restriction in the rhesus macaque attenuates markers of ovarian aging in select populations
Source: Aging (Albany NY). 2025 May 20;17(5):1164–89. doi: 10.18632/aging.206253 (PMC12151502; doi:10.18632/aging.206253)
Supplement: Supplementary Tables [file aging-17-206253-s002.pdf]

SUPPLEMENTARY TABLES

Supplementary Table 1. Statistical analysis of homogeneity of variation, sources of variation and main effects for follicle number per area as a function of diet and age.

		Source of variation		
**Follicle Class**	**Levene's test for homogeneity of variance**	**Interaction**	**Diet**	**Age**
Total	ns: $p = 0.1451$	ns: $p = 0.1815$	ns: $p = 0.1667$	$^{**}p = 0.0010$
Primordial	ns: $p = 0.9574$	ns: $p = 0.1592$	ns: $p = 0.2856$	$^{***}p = 0.0009$
Tr. Primordial	ns: $p = 0.639$	ns: $p = 0.2391$	ns: $p = 0.2145$	$^{***}p = 0.00005$
Primary	ns: $p = 0.7207$	ns: $p = 0.7258$	ns: $p = 0.4527$	$^{***}p = 0.0007$
Tr. Primary	ns: $p = 0.6891$	ns: $p = 0.4048$	ns: $p = 0.4350$	$^{**}p = 0.0090$
Secondary	ns: $p = 0.8815$	ns: $p = 0.2913$	ns: $p = 0.4619$	$^{***}p = 0.0003$
Multilayer	$^{*}0.0416$	ns: $p = 0.1736$	ns: $p = 0.4053$	$^{**}p = 0.0077$
Antral	ns: $p = 0.8419$	ns: $p = 0.9163$	ns: $p = 0.2701$	ns: $p = 0.0726$
Multi-oocytic	ns: $p = 0.2819$	ns: $p = 0.5965$	ns: $p = 0.9949$	$^{**}p = 0.0017$
Secondary with AMF	ns: $p = 0.9453$	ns: $p = 0.1755$	ns: $p = 0.1633$	ns: $p = 0.0654$
Multilayer with AMF	ns: $p = 0.6337$	ns: $p = 0.6040$	ns: $p = 0.8870$	ns: $p = 0.1440$
Atretic Antral	ns: 0.8485	ns: $p = 0.7445$	ns: $p = 0.7463$	$^{**}p = 0.0012$

Supplementary Table 2. Animal characteristics, including age at necropsy, cyclicity, and ovary weight for each individual animal in the study.

	Animal ID	Group	Age at necropsy (yrs)	Cyclicity	Ovary weight (mg)
Y-CON-1	22360	Young, Control	10	Regular	326
Y-CON-2	22362	Young, Control	13	Regular	578
Y-CON-3	22365	Young, Control	13	Regular	314
Y-CON-4	22367	Young, Control	13	Regular	314
O-CON-1	22342	Old, Control	26	Non-cycling	151
O-CON-2	22343	Old, Control	22	Irregular	377
O-CON-3	22345	Old, Control	26	Non-cycling	155
O-CON-4	22350	Old, Control	22	Irregular	200
O-CON-5	22351	Old, Control	22	Irregular	255
O-CON-6	22352	Old, Control	19	Regular	326
O-CON-7	22355	Old, Control	25	Irregular	340
O-CON-8	22368	Old, Control	25	Irregular	145
Y-CR-1	22357	Young, CR	13	Regular	400
Y-CR-2	22359	Young, CR	13	Regular	205
Y-CR-3	22361	Young, CR	13	Regular	360
Y-CR-4	22364	Young, CR	13	Regular	554
O-CR-1	22341	Old, CR	26	Irregular	226
O-CR-2	22347	Old, CR	25	Non-cycling	72
O-CR-3	22353	Old, CR	21	Irregular	210
O-CR-4	22354	Old, CR	20	Irregular	165
O-CR-5	22356	Old, CR	23	Irregular	125

Supplementary Table 3. Statistical analysis of homogeneity of variation, sources of variation and main effects for standardized follicle count (SFC) as a function of diet and age.

Follicle Class	Levene's test for homogeneity of variance	Source of variation: Interaction	Diet	Age
Total	ns: p = 0.1621	ns: p = 0.3015	ns: p = 0.2005	$^{***}p$ = 0.0005
Primordial	ns: p = 0.9553	ns: p = 0.2947	ns: p = 0.3586	$^{***}p$ = 0.0006
Tr. Primordial	ns: p = 0.5130	ns: p = 0.3298	ns: p = 0.2103	$^{***}p$ = 0.0003
Primary	ns: p = 0.7806	ns: p = 0.8938	ns: p = 0.5039	$^{***}p$ = 0.0004
Tr. Primary	ns: p = 0.4447	ns: p = 0.5693	ns: p = 0.4096	$^{**}p$ = 0.0039
Secondary	ns: p = 0.8815	ns: p = 0.7486	ns: p = 0.6251	$^{***}p$ = 0.0002
Multilayer	ns: p = 0.1038	ns: p = 0.3257	ns: p = 0.4218	$^{**}p$ = 0.0015
Antral	ns: p = 0.5314	ns: p = 0.6601	ns: p = 0.3867	$^{*}p$ = 0.0352
Multi-oocytic	ns: p = 0.5401	ns: p = 0.5215	ns: p = 0.9632	$^{**}p$ = 0.0012
Secondary with AMF	ns: p = 0.9874	ns: p = 0.4689	ns: p = 0.3682	$^{*}p$ = 0.0353
Multilayer with AMF	ns: p = 0.7935	ns: p = 0.8251	ns: p = 0.8936	ns: p = 0.0896
Atretic Antral	ns: p = 0.8685	ns: p = 0.9752	ns: p = 0.7993	$^{***}p$ = 0.0007

Supplementary Table 4. Raw follicle number data (mean/median/min/max) for young control, young calorie-restricted (CR), old control, and old calorie-restricted (CR) for all follicle classes (normal and abnormal).

		Primordial	Tr. Primordial	Primary	Tr. Primary	Secondary	Multilayer	Antral	Total	Secondary AMF	Multilayer AMF	Atretic Antral	Multi-oocytic
Young CON	Mean	232	448	24.3	50.8	29.8	38	6	828.8	14.3	10.8	24.3	15.3
	Median	201.5	387	21	40.5	28	39.5	5	727	14	12	23.5	13
	Min.	30	105	11	25	14	15	0	221	0	2	2	6
	Max.	495	913	44	97	49	58	14	1640	29	17	48	29
Young CR	Mean	209	490.8	35.5	59.3	26.3	28.5	12.8	862	28.5	10.8	19.3	12.3
	Median	241.5	590	31	65.5	28	23	11.5	1037	15	12.5	17.5	12
	Min.	2	21	7	4	5	6	1	48	0	0	1	2
	Max.	351	762	73	102	44	62	27	1326	84	18	41	23
Old CON	Mean	9.4	26	4.8	8.7	3	4.9	1.8	58.4	0.875	1.875	6.5	1.8
	Median	0.5	1.5	0	0.5	0	1.5	0.5	5.5	0	0	2.5	1
	Min.	0	0	0	0	0	0	0	0	0	0	0	0
	Max.	69	176	31	40	14	16	7	351	4	7	28	5
Old CR	Mean	13.8	31.4	2.8	9	3.2	5.8	1.2	67.2	0.2	3.6	7.8	1.6
	Median	13	21	2	8	4	6	1	59	0	3	3	0
	Min.	0	1	0	0	0	1	0	7	0	0	0	0
	Max.	28	87	7	19	6	10	2	153	1	7	25	5

Supplementary Table 5. Statistical analysis of homogeneity of variation, sources of variation and main effects for follicle number per area as a function of diet and menstrual cyclicity.

		Source of variation		
**Follicle class**	**Levene's test for homogeneity of variance**	**Interaction**	**Diet**	**Menstrual cyclicity**
Total	ns: $p = 0.6020$	ns: $p = 0.1831$	ns: $p = 0.708$	$^{***}p = 0.0007$
Primordial	ns: $p = 0.8660$	ns: $p = 0.1527$	ns: $p = 0.1339$	$^{***}p = 0.0006$
Tr. Primordial	ns: $p = 0.9522$	ns: $p = 0.2370$	ns: $p = 0.0864$	$^{***}p = 0.0003$
Primary	ns: $p = 0.1314$	ns: $p = 0.4250$	ns: $p = 0.2130$	$^{***}p = 0.0001$
Tr. Primary	ns: $p = 0.6412$	ns: $p = 0.4304$	ns: $p = 0.3076$	$^{**}p = 0.0047$
Secondary	ns: $p = 0.7078$	ns: $p = 0.2215$	ns: $p = 0.2396$	$^{***}p = 0.0001$
Multilayer	ns: $p = 0.5879$	ns: $p = 0.1286$	ns: $p = 0.2682$	$^{**}p = 0.0088$
Antral	ns: $p = 0.9515$	ns: $p = 0.9224$	ns: $p = 0.2219$	ns: $p = 0.976$
Multi-oocytic	ns: $p = 0.3765$	ns: $p = 0.9427$	ns: $p = 0.7696$	$^{**}p = 0.0033$
Secondary with AMF	ns: $p = 0.7987$	ns: $p = 0.2253$	ns: $p = 0.1245$	ns: $p = 0.691$
Multilayer with AMF	ns: $p = 0.1052$	ns: $p = 0.6450$	ns: $p = 0.7790$	ns: $p = 0.2250$
Atretic Antral	ns: $p = 0.1153$	ns: $p = 0.6691$	ns: $p = 0.9526$	$^{**}p = 0.0021$

Supplementary Table 6. Statistical analysis of homogeneity of variation, sources of variation and main effects for standardized follicle count (SFC) as a function of diet and menstrual cyclicity.

		Source of variation		
**Follicle class**	**Levene's test for homogeneity of variance**	**Interaction**	**Diet**	**Menstrual cyclicity**
Total	ns: $p = 0.6020$	ns: $p = 0.1831$	ns: $p = 0.708$	$^{***}p = 0.0007$
*P*rimordial	ns: $p = 0.8660$	ns: $p = 0.1527$	ns: $p = 0.1339$	$^{***}p = 0.0006$
Tr. *P*rimordial	ns: $p = 0.9522$	ns: $p = 0.2370$	ns: $p = 0.0864$	$^{***}p = 0.0003$
*P*rimary	ns: $p = 0.1314$	ns: $p = 0.4250$	ns: $p = 0.2130$	$^{***}p = 0.0001$
Tr. *P*rimary	ns: $p = 0.6412$	ns: $p = 0.4304$	ns: $p = 0.3076$	$^{**}p = 0.0047$
Secondary	ns: $p = 0.7078$	ns: $p = 0.2215$	ns: $p = 0.2396$	$^{***}p = 0.0001$
Multilayer	ns: $p = 0.5879$	ns: $p = 0.1286$	ns: $p = 0.2682$	$^{**}p = 0.0088$
Antral	ns: $p = 0.9515$	ns: $p = 0.9224$	ns: $p = 0.2219$	ns: $p = 0.976$
Multi-oocytic	ns: $p = 0.3765$	ns: $p = 0.9427$	ns: $p = 0.7696$	$^{**}p = 0.0033$
Secondary with AMF	ns: $p = 0.7987$	ns: $p = 0.2253$	ns: $p = 0.1245$	ns: $p = 0.691$
Multilayer with AMF	ns: $p = 0.1052$	ns: $p = 0.6450$	ns: $p = 0.7790$	ns: $p = 0.2250$
Atretic Antral	ns: $p = 0.1153$	ns: $p = 0.6691$	ns: $p = 0.9526$	$^{**}p = 0.0021$
